# *Myxobolus dabryi* n. sp. (Myxozoa: Myxobolidae) Infecting the Gills of *Chanodichthys dabryi,* Bleeker, 1871 (Cypriniformes: Cyprinidae) in Hunan Province, China †

**DOI:** 10.3390/ani14172487

**Published:** 2024-08-27

**Authors:** Xiaojing Zhao, Qi Yin, Jia Cai, Qiang Wei, Deliang Li, Jianbo Yu, Jianguo Xiang, Jinyong Zhang, Xinhua Liu

**Affiliations:** 1 Hunan Engineering Research Center for Utilization of Characteristics of Aquatic Resources, Hunan Agricultural University, Changsha 410128, China; zhaoxiaojing23@126.com (X.Z.); yq01266369@stu.hunau.edu.cn (Q.Y.); lidl@hunau.edu.cn (D.L.);; 2 School of Marine Science and Engineering, Qingdao Agricultural University, Qingdao 266109, China; zhangjy@ihb.ac.cn; 3 Guangdong Provincial Key Laboratory of Aquatic Animal Disease Control and Healthy Culture, Zhanjiang 524000, China

**Keywords:** *Myxobolus*, gill arch, Culters, phylogeny

## Abstract

**Simple Summary:**

Myxozoa is an important metazoan parasite, with approximately 2600 species reported around the world. However, their diversity is still largely underestimated, particularly for those of the Culters, where only 15 species have been previously reported in China. Thus, an investigation into the diversity of myxozoans in Culters was conducted, and a new species belonging to *Myxobolus* was found. Through comprehensive morphological comparisons and molecular analyses with closely related species, we designated it a new *Myxobolus* species and named it *Myxobolus darbryi* n. sp.

**Abstract:**

Culters are a popular and economically important carnivorous freshwater fish, widely distributed in rivers, lakes, and reservoirs in China. An investigation of Myxozoa was conducted to enhance the understanding of Myxozoan diversity in Culters in China, as only 15 Myxosporean species have been previously reported in 6 Culters species. A new species with typical *Myxobolus* characteristics was discovered exclusively in the gills of *Chanodichthys dabryi*, Bleeker, 1871, and no other species were found in other Culters fish or organs. The new species elicited whitish plasmodia in the serosa layer of the gill arch, with no distinct inflammatory reaction observed. This species is morphologically different from all reported *Myxobolus* spp. from Culters, differing in plasmodium and spore size, as well as the coils of polar filaments. Molecular analysis further supports that it does not match any sequences available in GenBank. Therefore, we identified it as a new species and named it *Myxobolus dabryi* n. sp.

## 1. Introduction

Myxozoa are an important metazoan parasite in aquaculture, with approximately 2600 species reported from various organs or tissues of different hosts (mainly fish) [1,2,3]. Among them, more than 700 species have been described in China [4,5]. Most of them were asymptomatic and harmless to their host; however, a few species can cause serious myxosporidiosis, leading to significant economic losses. Notable examples include the pharyngeal myxosporidiosis caused by *Myxobolus honghuensis* Liu, Whipps, Gu, Zeng & Huang, 2012 from *Carassius auratus gibelio* Bloch, 1782, liver myxosporidiosis caused by *Myxobolus wulii* Lansberg & Lom, 1991 also from *C. auratus gibelio*, skin myxosporidiosis caused by *Thelohanellus wuhanensis* Xiao & Chen, 1993 again from *C. auratus gibelio*, and intestinal giant-cystic disease caused by *Thelohanellus kitauei* Egusa & Nakajima, 1981 from scattered mirror carp (*Cyprinus carpio* Linnaeus, 1758) [4,6,7,8,9].

*Chanodichthys dabryi* Bleeker, 1871 (Cyprinidae: Xenocypridinae) is an important carnivorous fish [10]. They are renowned for their delicious meat, strong resistance to disease, and high economic value, and are widely distributed in rivers (such as the Yangtze River), lakes (such as the Lake Poyang), and reservoirs (such as Danjiangkou reservoir) in China [10]. Currently, only seven myxosporean species have been previously documented in *C. dabryi*. These include four celozoic species (gallbladder-infecting): *Myxidium polymorphum* Nie & Li, 1973, *Chloromyxum sinensis* Nie & Li, 1992, *Chloromyxum ellipticum* Li & Nie, 1973, and *Chloromyxum hupehensis* Li & Nie, 1973, as well as three histozoic species (gill-infecting): *Myxobolus chengkiangensis* Ma, 1998, *Myxobolus erythroculteri* Nie & Li, 1992, and *Myxobolus chuhsienensis* Chen & Ma, 1998. No species of *Thelohanellus* Kudo, 1933 or *Henneguya* Thélohan, 1892 have been previously documented in this host, although they are frequently found in freshwater fish in China [4]. Due to historical and technological limitations, these seven species were described based solely on morphological characteristics, which are not compatible with the current taxonomic methods for Myxozoa that integrate morphological, ecological (host specificity and tissue tropism), and molecular characteristics [1,4,11,12]. Thus, to elucidate the diversity of Myxozoa of *C. dabryi* in China, an investigation was conducted, resulting in the discovery and identification of a new species, *Myxobolus dabryi* n. sp., based on morphological, histopathological, and molecular characteristics.

## 2. Materials and Methods

### 2.1. Morphological Examination

Ten specimens of *C. dabryi*, ranging from 25.7 to 27.2 cm in body length, were randomly collected in March 2023 from Lake Datong, Hunan Province, China. The fish were placed alive in a 5 L simple tank and immediately transported to the laboratory. Initially, the fish were euthanized using an overdose of tricaine methane sulfonate (MS-222). A comprehensive inspection of various organs including the gills, skin, fins, liver, gallbladder, kidney, spleen, and intestine, was conducted to identify suspected plasmodia (generally whitish cysts) visible to the naked eyes or a Motic SMZ161 dissecting microscope (Motic, Beijing, China). Once found, the plasmodia were used to prepare microscope slides for further examination under an Olympus BX53 microscope (Olympus, Nagano, Japan) at 400× magnification. These slides were also used to capture photos of fresh spores with the Olympus BX53 microscope, equipped with an MSX 11 digital camera (Mingmei, Guangzhou, China), at 400× magnification. The morphological features of the spores and plasmodia were obtained from the digital images according to the guidelines described by Lom and Arthur [13]. All measurements are recorded in μm as means ± standard error (range) (*n* = 50). Schematic drawings of spores were created using CorelDraw X6 software (Corel, Ottawa, ON, Canada) based on the photos taken above.

### 2.2. Histopathological Examination

After fixing in 10% neutral buffered formalin for 24 h, the infected gills were dehydrated in a series of ethanol, embedded in paraffin wax, sectioned at 5–6 μm, stained with hematoxylin and eosin (H&E), and finally observed and photographed under light microscopy.

### 2.3. Molecular Characterization

#### 2.3.1. DNA Isolation and Sequencing

One sample of four isolated plasmodia preserved in 70% ethanol was randomly selected and subjected to DNA extraction using a Qiagen DNeasy Blood&Tissue Kit (Qiagen, Hilden, Germany) as described previously by Liu et al. [11]. The partial nuclear 18S rDNA gene sequences were amplified using the MyxospecF [14] and 18R [15] primer pair, with an expected length of approximately 1800 base pairs. PCR were performed in a 25 µL reaction mixture that contained 100 ng of extracted template DNA, 1 × PCR mixture (Vazyme Biotech, Nanjing, China), and 10 pmol of each primer. The thermocycler parameters comprised an initial denaturation step at 94 °C for 4 min, followed by 35 cycles of 94 °C for 1 min, 46 °C for 50 s, and 65 °C for 90 s; with a final extension at 65 °C for 10 min. PCR products were analyzed by agarose gel electrophoresis and purified using a DNA Gel Extraction kit (CWBiotech, Taizhou, China). The purified DNA template was cloned into the PMD–18T vector (Takara, Kusatsu, Japan) according to the manufacture’s recommended instructions and transformed into *Escherichia coli* (*E. coli*) DH5α competent cell. Three positive recombinant clones were selected and subjected to sequence on both sides on an ABI PRISM 3100 automatic sequencer (Applied Biosystems, Beijing, China).

#### 2.3.2. Phylogenetic Analysis

The sequences were aligned in BioEdit [16] and ambiguous bases were clarified with the corresponding ABI chromatograms. The consensus sequence was deposited in GenBank under accession number OP359407 and verified as myxozoan based on the BLAST search. To explore the phylogenetic relationships of the novel species with related myxobolids, 40 myxosporean sequences (Table 1), including those most similar based on the BLASTN result and others of interest, were retrieved from GenBank and aligned using the Clustal X 1.8 program with the default setting [17]. The alignment was corrected manually using the alignment editor within MEGA 11.0.13 software [18]. Phylogenetic analyses were inferred using Bayesian inference (BI) and Maximum Likelihood (ML), which were carried out by PhyML 3.0 and Mr. Bayes, respectively [19,20]. The optimal evolutionary model for ML and BI was GTR + F + I + G4, as determined by jModelTest 3.0 using the Akaike information criteria (AIC) [21]. Nucleotide frequencies were estimated from the data (A = 0.267, C = 0.196, G = 0.282, T = 0.255); nucleotide substitution rates were calculated as AC = 0.9490, AG = 2.9064, AT = 1.2381, CG = 0.7829, CT = 4.5860, GT = 1.0000. The proportion of the invariable site was 0.268 and the alpha value of the gamma distribution parameter was 0.380. *Myxidium finnmarchicum* Mackenzie, Collins, Kalavati & Hemmingsen, 2010 (GQ890673) was used as the outgroup. A total of 2 independent runs with 4 chains were conducted for 5 million generations for BI, with a sample frequency of 100, discarding the first 25% of trees as burn-in. Bootstrap supports were calculated from 1000 repetitions for ML. The generated phylogenetic tree was visualized using FigTree v1.4.2 [22], edited and annotated with Adobe Illustrator (Adobe Systems Inc., San Jose, CA, USA).

## 3. Results

**Figure 1:**
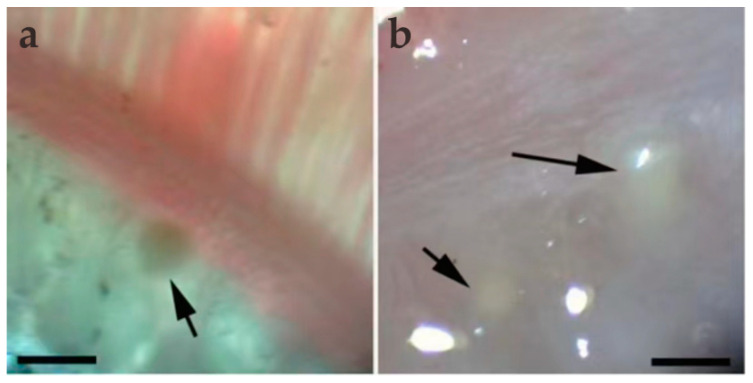
Light microscopic observation of *M. dabryi* n. sp. of *Chanodichthys dabryi*. (**a**) A plasmodium dwelled (arrow) in the gill arches, scale bar = 500 µm; (**b**) two macroscopically visible whitish plasmodia (arrow) in the gill arches, scale bar = 500 µm.

During sampling, 10 *C. dabryi* were dissected to investigate myxosporean infections, and plasmodia were exclusively found in the gill arch (Figure 1). The prevalence of *M. dabryi* n. sp. was 20% (2/10). The plasmodia (n = 10) were ellipsoidal and measured 0.63–0.68 mm long and 0.31–0.36 mm wide. No plasmodium or mature spores were found in other organs such as the intestine, kidney, spleen, skin, or liver.

### Myxobolus dabryi n. sp. (Figure 2, Figure 3, Figure 4 and Figure 5, Table 2)

**Figure 2:**
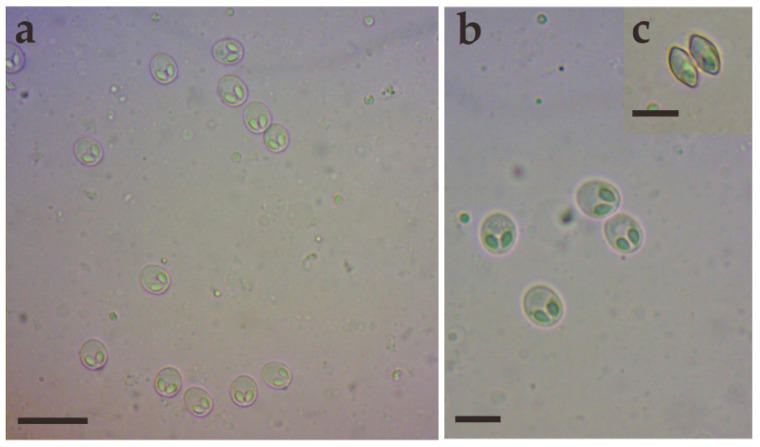
Fresh spores of *Myxobolus dabryi* n. sp. infecting the gills of *Chanodichthys dabryi*. (**a**) Fresh spores, scale bar = 20 µm; (**b**) four fresh spores in the frontal view, scale bar = 10 µm; (**c**) fresh spores in the sutural view, scale bar = 10 µm.

**Figure 3:**
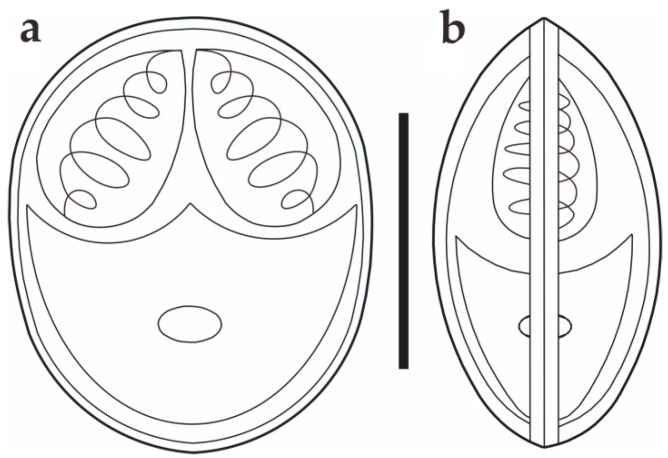
Schematic drawings of *M. dabryi* n. sp. from *Chanodichthys dabryi*, spore in the frontal view (**a**) and in the sutural view (**b**), scale bar = 10 µm.

Morphological description: Fresh spores (n = 50) were ellipsoidal in the frontal view and fusiform in the sutural view, measuring 9.2 ± 0.4 (8.3–9.9) μm long, 7.4 ± 0.3 (6.9–8.0) μm wide and 5.2 ± 0.2 (4.8–5.5) μm thick. The surface of the spore was smooth, and the valves of the spore were symmetric. Two equal polar capsules were pyriform, averaging 3.5 ± 0.1 (3.3–3.7) μm long and 2.1 ± 0.1 (2.0–2.4) μm wide. The polar filaments were coiled with four to five turns, occupying about half the length of the spore. The mucous envelope, iodinophilous vacuoles, intercapsular appendix, *Henneguya*-like spores, and posterior folds were not observed in the present study (Figure 2 and Figure 3).

**Figure 4:**
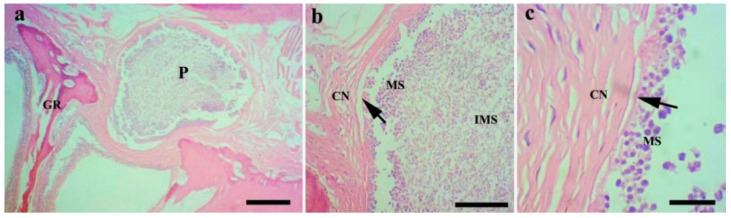
Histopathological analysis of the gills of *Chanodichthys dabryi* infected with *M. dabryi* n. sp. (**a**) The plasmodium (P) located in the serosa layer of the gill arch (GR), scale bar = 200 µm; (**b**) the magnification of the plasmodium in (**a**), indicating the plasmodium surrounded by connective tissues (CN) with mature spores (MS) located centrally and immature spores (IMS) peripherally, scale bar = 50 µm; (**c**) the magnification of the periphery of the plasmodium in (**b**), showing the plasmodium compressed slightly to adjacent connective tissues (arrow), scale bar = 10 µm.

Histological analysis (n = 8) showed that the *M. dabryi* n. sp. plasmodia resided in the serosa layer of the gill arches (Figure 4). The plasmodium was surrounded by a layer of connective tissues, with mature spores accumulated in the periphery and immature spores located centrally (Figure 4). No distinct inflammatory reaction was observed in the infection focus.

**Figure 5:**
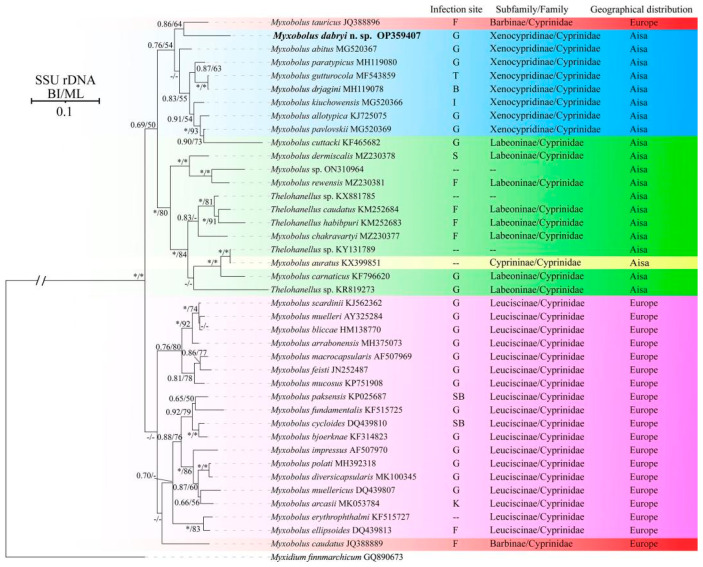
Phylogenetic tree of *M. dabryi* n. sp. and its relatives based on the SSU rDNA sequences, rooted at *M. finnmarchicum*. Support values on the branch nodes are indicated as BI posterior probabilities/ML support values; asterisks represent support values > 0.95 or 95% and dashes demonstrate support values < 0.50 or 50%; abbreviations: B: brain; F: fins; G: gills; I: intestine; K: kidney; P: pin bones; S: scales; SB: swim bladder; T: throat; --: unknown.

Molecular characterization: The SSU rDNA gene sequence of *M. dabryi* n. sp. was 1728 bp and contained 50.06% GC bases. BLASTn demonstrated that it did not match any sequences available in GenBank but was most closely related to *Myxobolus abitus* Li & Nie, 1973 (92.17% over 1736 bp, MG520367) infecting the gills of silver carp *Hypophthalmichthys molitrix* Valenciennes, 1844. Other similar sequences included *Myxobolus tauricus* Miroshnichenko, 1979 (91% over 1424 bp, JQ388896) infecting the fins of the common barbel *Barbus barbus* Linnaeus, 1758, *Myxobolus kiuchowensis* Chen, 1998 (91.12% over 1734 bp, MG520366) from the intestine of bighead carp *Hypophthalmichthys nobilis* Richardson, 1845, *Myxobolus pavlovskii* Akhmerov, 1954 (90.98% over 1741 bp, MG520369) from the gills of *H. molitrix* and *Myxobolus mucosus* Liu, Voronin, Dudin & Zhang, 2015 (90.49% over 1746 bp, KP751908) from the gills of *Rultilus rutilus* Linnaeus, 1758 and *Leuciscus leuciscus* Linnaeus, 1758. The pairwise distances and similarities calculated using Kimura 2-parameter model among the present species and those with high sequence similarity ranged from 0.100/90.0% to 0.066/93.4% (Table 2). ML and BI analysis produced a similar topological tree, although with somewhat different support values at some evolutionary nodes. Therefore, only the Bayesian tree was presented here, along with the bootstrap values of ML analysis. Phylogenetic analyses were carried out based on a final edited alignment of 1109 characters, which indicated that the present species initially clustered with *M. tauricus* and then formed a sister group to the aforementioned species with high sequence similarity within the group of Xenocypridinae-infecting *Myxobolus* Bütschli, 1882 species with moderate supported values (Figure 5).

**Table 2:** Comparison of sequence similarities (above diagonal) and genetic distances (below diagonal) of *Myxobolus dabryi* n. sp. with other genetically related *Myxobolus* spp. based on the partial 18S rDNA.

Species	1	2	3	4	5	6	7	8	9	10	11	12	13
1 *M. dabryi* n. sp. OP359407		93.4	93.3	92.1	91.4	91.3	91.3	91.2	91.1	91.1	90.9	90.9	90.0
2 *M. tauricus* JQ388896	0.066		95.4	94.8	91.9	91.2	91.1	92.3	91.5	91.2	91.5	91.7	90.0
3 *M. abitus* MG520367	0.067	0.046		96.0	90.8	92.8	92.8	92.0	92.5	91.4	92.6	91.5	90.5
4 *M. drjagini* MW577455	0.079	0.052	0.040		91.3	91.3	91.3	92.1	92.0	90.2	92.0	91.0	90.2
5 *M. erythrophthalmi* KF515728	0.086	0.081	0.092	0.087		92.5	92.6	95.7	93.7	90.5	93.6	92.4	91.5
6 *M. scardinii* KJ562362	0.087	0.088	0.072	0.087	0.075		99.6	93.5	93.4	90.5	93.3	0.919	0.914
7 *M. bliccae* HM138770	0.087	0.089	0.072	0.087	0.074	0.004		93.5	93.4	90.8	93.3	92.0	91.5
8 *M. zaikae* MT141124	0.088	0.077	0.080	0.079	0.043	0.065	0.065		94.1	90.5	93.8	92.9	91.6
9 *M. polati* MH392318	0.089	0.085	0.075	0.080	0.063	0.066	0.066	0.059		91.6	99.5	95.6	94.5
10 *M. chakravartyi* MZ230377	0.089	0.088	0.086	0.098	0.095	0.095	0.092	0.095	0.084		91.3	90.3	89.5
11 *M. diversicapsularis* GU968199	0.091	0.085	0.074	0.080	0.064	0.067	0.067	0.062	0.005	0.087		95.8	94.9
12 *M. impressus* AF507970	0.091	0.083	0.085	0.090	0.076	0.081	0.080	0.071	0.044	0.097	0.042		96.1
13 *M. lamellobasis* KF314824	0.100	0.100	0.095	0.098	0.085	0.086	0.085	0.084	0.055	0.105	0.051	0.039	

Taxonomic Summary

Species: *Myxobolus dabryi* n. sp.

Type host: *Chanodichthys dabryi* (Bleeker, 1871) (Cypriniformes: Cyprinidae).

Type locality: Lake Datong, Hunan province, China (29°20′37″ N, 112°50′77″ E).

Site of infection: Gills.

Prevalence: 20% (2/10).

Type material: Syntype specimens were fixed in 10% formalin and 95% ethanol, H & E-stained tissue section, and their digitized photos were deposited in the Fishes Diseases Laboratory, College of Fisheries, Hunan Agricultural University (accession number MTR2023031901-MTR2023031906).

Etymology: The species is named after the species name of the host.

ZoonBank number: urn:lsid:zoobank.org:pub:1B6D1B6C-18CC-4DCE-AB53-4B7211D5C68B.

## 4. Discussion

In China, six Culters species have previously been found in different rivers or lakes, including *Culter alburnus* Basilewsky, 1855, *Chanodichthys dabryi* Bleeker, 1871, *Chanodichthys erythropterus* Basilewsky, 1855, *Chanodichthys mongolicus* Basilewsky, 1855, *Chanodichthys oxycephalus* Bleeker, 1871, and *Culter oxycephaloides* Kreyenberg & Pappenheim, 1908 [10]. Additionally, only 15 myxosporean species have been reported, including 7 *Myxobolus* spp., 4 *Chloromyxum* spp., 1 *Myxidium* sp., 1 *Henneguya* sp., 1 *Sphaerospora* sp., and 1 *Podospora* sp. [4]. Among them, five *Myxobolus* species were most morphologically similar to *M. dabryi* n. sp., which included three *C. dabryi* gill-infecting *Myxobolus* spp. (*M. chuhsienensis*, *Myxobolus erthroculteri* Nie & Li, 1992, and *Myxobolus changjiangensis* Ma, 1993),along with *Myxobolus lussi* Akhmerov, 1960 from the gills of *C. mongolicus*, and *Myxobolus dermatobia* Ishii, 1915 infecting the kidney of *C. alburnus* [4]. *M. dabryi* n. sp. could be easily differentiated from all of them by difference in spore shapes, infection sites, spore sizes, etc. *M. chuhsienensis* is distinguished from the present species by its smaller cysts (0.21–0.25 mm × 0.12–0.17 mm vs. 0.63–0.68 mm × 0.31–0.36 mm), as well as shorter spore length (7.2–8.7 μm vs. 8.3–9.9 μm) and width (6.7–7.0 μm vs. 6.9–8.0 μm). Furthermore, three to four folds could be observed in the posterior of *M. chuhsienensis*, which are absent in the present species. The spores of *M. erthroculteri* and *M. changjiangensis* are pyriform, differing from the ellipsoidal spores of *M. dabryi* n. sp. Additionally, *M. erthroculteri* has a larger spore length (12.5–14.0 μm vs. 8.3–9.9 μm), thicker spore thickness (6.0–7.5 μm vs. 4.8–5.5 μm), and two larger polar capsules. *M. changjiangensis* is differentiated from *M. dabyri* n. sp. by its larger spores (26.5–28.0 × 21.0–22.0 × 15.0 μm vs. 8.3–9.9 × 6.9–8.0 × 4.8–5.5 μm) and polar capsules (12.0–14.0 × 6.0–7.0 μm vs. 3.3–3.7 × 2.0–2.4 μm). *M. lussi* and *M. dermatobia* resemble *M. dabryi* n. sp. in having an ellipsoidal spore shape. However, *M. lussi* can be differentiated from *M. dabryi* n. sp. by its larger whitish cysts (1.5–2 mm vs. 0.63–0.68 × 0.31–0.36 mm), apparent intercapsule appendix, longer spore length (10.6–12.0 μm vs. 8.3–9.9 μm), and more turns of polar filaments (6–7 vs. 4–5). *M. dermatobia* is distinguished from *M. dabryi* n. sp. by differing sites of infection (kidney vs. gills), slightly shorter spore length (7.4–8.4 μm vs. 8.3–9.9 μm), and narrower spore width (6.0–7.2 μm vs. 6.9–8.0 μm) (Table 3).

The gills located on both sides of the fish head are vital to fish due to their various physiological functions, including respiration (the major function), ammonia nitrogen excretion, filter feeding, etc. [23]. *Myxobolus* Bütschli, 1882 is the speciose genus within Myxozoa, with more than 1000 species reported worldwide. Almost half of them inhabit different portions of the gills, including the gill filaments, gill lamellae, gill arch, and gill rakers [5,13,24,25,26]. Gill myxosporidiosis mainly occurs in species dwelling in the gill filaments or gill lamellae (such as *Myxobolus koi* Kudo, 1920 from *C. carpio* and *Myxobolus ampullicapsulatus* Zhao, Sun, Kent, Deng & Whipps, 2008 from *C. auratus gibelio*) [27,28]. No distinct damage has been found in *Myxobolus* spp. infecting the gill arch. Currently, the *Myxobolus* species described in the gill arch can be divided into three categories: those dwelling in the epithelial tissues or connective tissues (most of them), those in the cartilaginous substances, and those in blood vessels [26,29,30]. The plasmodium of *M. dabryi* n. sp. is similar to *Myxobolus niger* Mathews, Maiab & Adriano, 2016, which is located in the serosa layer of the gill arch and is surrounded by a layer of connective tissues. This plasmodium causes slight compression to the adjacent tissues, but no distinct inflammatory reaction has been observed.

Phylogenetic analysis indicated that the selected species were clustered together mainly according to geographical distribution (Asia group and Europe group) and host subfamily (Xenocypridinae group, Labeoninae group, and Leuciscinae group) with few exceptions. Currently, numerous studies have been conducted to assess the impact of host affinity, tissue tropism, and spore shapes on the evolutionary routes of Myxozoa, but little attention has been paid to the role of geographic distribution [31]. This may be due to the difficulties in evaluating its influence, especially concerning species of invasive fish. For example, *M. pavlovskii* infecting the gills of silver carp *H. molitrix* and bighead carp *H. nobilis* and *Thelohanellus nikolskii* Achmerov, 1955 infecting the fins of common carp *C. carpio* L., have been reported in both China (Asia) and Hungary (Europe) [31,32,33,34,35]. Several myxosporean species have also been discovered in invasive fish in China, such as *Cirrhinus mrigala* Hamilton, 1822, *Labeo rohita* Hamilton, 1822, and *Luciobarbus brachycephalus* Kessler, 1872 [36,37]. It is difficult to determine whether these species are endemic to the importing country or whether they were introduced alongside other fish. Furthermore, some natural water bodies, such as the Amur River, are distributed across three countries, China (Asia), Mongolia (Asia) and Russia (Europe), leading to the presence of many species found in both Asia (China and Mongolia) and Europe (Russia) [4]. This complicates the evaluation of the role of geographic distribution in the evolution of myxozoa.

The infection site is an important taxonomic feature of Myxozoa; different species infect specific sites or several different sites [25]. The present species did not cluster with gill-infecting *Myxobolus* species, but clustered with *M. tauricus* infecting the fin and pin bones of *Barbus barbus* within the Xenocypridinae group. This group included species that infect various organs, including the gills, brain, intestine, and throat. This finding does not agree with previous research indicating that *Myxobolus* species primarily group with each other based on infection site tropism [31,38]. Shin et al. [31] demonstrated that the infection site tropism could be divided into specific infection-site tropism (infecting the gills or muscles) and non-specific infection-site tropism (affecting almost all organs). The case of *M. dabryi* n. sp. resembles those species with non-specific infection site tropism, while the species in the Europe group species exhibit specific infection-site tropism. Host specificity, site tropism, and spore shapes are important features for the taxonomy of Myxozoa and are correlated with the phylogeny of the group. Zhang et al. [12] indicated that host species possess a stronger evolutionary signal than spore morphology and site tropism, which aligns with the findings herein and other previously reported studies [36,38]. Therefore, we speculate that the host may be the primary selective pressure, followed by site infections and spore morphology. The diversity of spore morphology in Myxozoa may result from the long-term co-evolution of interactions between hosts (including different host and different site infections) and parasites. However, further research is needed to verify this hypothesis.

## 5. Conclusions

In summary, a new species, *M. dabryi* n. sp. infecting the gill arch of *C. dabryi,* has been comprehensively described using morphological, ecological, and molecular data.

## Figures and Tables

**Table 1:** The details of the selected sequences used in the phylogenetic analysis.

Species Name	Accession Number	Host	Infected Tissue	Source
*Myxobolus dabryi* n. sp.	OP359407	*Chanodichthys dabryi*	Gills	Present study
*Myxobolus abitus*	MG520367	*Hypophthalmichthys molitrix*	Gills	GenBank
*Myxobolus allotypica*	KJ725075	*Hypophthalmichthys molitrix*	Gills	GenBank
*Myxobolus arcasii*	MK053784	*Achondrostoma arcasii*	Kidney	GenBank
*Myxobolus arrabonensis*	MH375073	*Chondrostoma angorense*	Gills	GenBank
*Myxobolus auratus*	KX399851	*Carassius auratus auratus*	Unknown	GenBank
*Myxobolus bjoerknae*	KF314823	*Blicca bjoerkna*	Gills	GenBank
*Myxobolus bliccae*	HM138770	*Blicca bjoerkna*	Gills	GenBank
*Myxobolus carnaticus*	KF796620	*Cirrhinus mrigala*	Gills	GenBank
*Myxobolus caudatus*	JQ388889	*Barbus barbus*	Fins	GenBank
*Myxobolus chakravartyi*	MZ230377	*Labeo catla*	Fins	GenBank
*Myxobolus cuttacki*	KF465682	*Labeo bata*	Gills	GenBank
*Myxobolus cycloides*	DQ439810	*Squalius cephalus*	Swim bladder	GenBank
*Myxobolus dermiscalis*	MZ230378	*Labeo rohita*	Scales	GenBank
*Myxobolus diversicapsularis*	MK100345	*Rutilus rutilus*	Gills	GenBank
*Myxobolus drjagini*	MH119078	*Hypophthalmichys molitrix*	Brain	GenBank
*Myxobolus ellipsoides*	DQ439813	*Squalius cephalus*	Fins	GenBank
*Myxobolus erythrophthalmi*	KF515727	*Scardinius erythrophthalmus*	Unknown	GenBank
*Myxobolus feisti*	JN252487	*Rutilus rutilus*	Gills	GenBank
*Myxobolus fundamentalis*	KF515725	*Rutilus rutilus*	Gills	GenBank
*Myxobolus gutturocola*	MF543859	*Hypophthalmichthys molitrix*	Throat	GenBank
*Myxobolus impressus*	AF507970	*Abramis brama*	Gills	GenBank
*Myxobolus kiuchowensis*	MG520366	*Hypophthalmichthys nobilis*	Intestine	GenBank
*Myxobolus macrocapsularis*	AF507969	*Abramis brama*	Gills	GenBank
*Myxobolus mucosus*	KP751908	*Leuciscus leuciscus*	Gills	GenBank
*Myxobolus muelleri*	AY325284	*Squalius cephalus*	Gills	GenBank
*Myxobolus muellericus*	DQ439807	*Squalius cephalus*	Gills	GenBank
*Myxobolus paksensis*	KP025687	*Chondrostoma nasus*	Swim bladder	GenBank
*Myxobolus paratypicus*	MH119080	*Hypophthalmichys molitrix*	Gills	GenBank
*Myxobolus pavlovskii*	MG520369	*Hypophthalmichthys nobilis*	Gills	GenBank
*Myxobolus polati*	MH392318	*Chondrostoma angorense*	Gills	GenBank
*Myxobolus rewensis*	MZ230381	*Cirrhinus mrigala*	Fins	GenBank
*Myxobolus scardinii*	KJ562362	*Scardinius erythrophthalmus*	Gills	GenBank
*Myxobolus* sp.	ON310964	Unknown	Unknown	GenBank
*Myxobolus tauricus*	JQ388896	*Barbus barbus*	Fins	GenBank
*Thelohanellus caudatus*	KM252684	*Labeo rohita*	Fins	GenBank
*Thelohanellus habibpuri*	KM252683	*Labeo rohita*	Fins	GenBank
*Thelohanellus* sp.	KR819273	*Labeo catla*	Gills	GenBank
*Thelohanellus* sp.	KX881785	Unknown	Unknown	GenBank
*Thelohanellus* sp.	KY131789	Unknown	Unknown	GenBank
*Myxidium finnmarchicum*	GQ890673	*Gadus morhua*	gall bladder	GenBank

**Table 3:** Morphological comparisons of *Myxobolus dabryi* n. sp. with its similar species from Culters (all measurements present in micrometer).

Species	IS	H	S	LS	WS	TS	LPC	WPC	CPF	Ref
*M. dabryi* n. sp.	Gills	*C. dabryi*	Ellipsoide	9.2 ± 0.4 ^a^	7.4 ± 0.3	5.2 ± 0.2	3.5 ± 0.1	2.1 ± 0.1	4–5	Present study
				(8.3–9.9) ^b^	(6.9–8.0)	(4.8–5.5)	(3.3–3.7)	(2.0–2.4)		
*M. chuhsienensis*	Gills	*C. dabryi*	Ellipsoide	8.2 ^c^	7.0	5.3	3.5	2.3	?	[4]
				(7.2–8.7)	(6.7–7.0)	(5.0–5.6)	(3.5–3.7)	(2.1–2.5)		
*M. erythroculteri*	Gills	*C. dabryi*	Pyriform	12.5–14.0	7.4–9.0	6.0–7.5	6.0–7.5	2.4–3.0	?	[4]
*M. changjiangensis*	Gills	*C. dabryi*	Pyriform	26.5–28.0	21.0–22.0	15.0	12.0–14.0	6.0–7.0	4–5	[4]
*M. lussi*	Gills	*C. mongolicus*	Round	11.4	8.1	5.8	4.9	3.1	6–7	[4]
				(10.6–12.0)	(7.4–8.4)	(4.8–6.2)	(4.6–5.2)	(2.8–3.6)		
*M. dermatobia*	Kidney	*C. erythropeterus*	Ellipsoide	8.1	6.8	4.9	3.6	2.3	?	[4]
				(7.4–8.4)	(6.0–7.2)	(4.8–5.0)	(3.5–3.8)	(2.0–2.4)		

IS: site of infection, H: host, S: shape, LS: length of the spore, WS: width of the spore, TS: thickness of the spore, LPC: length of the polar capsule, WPC: width of the polar capsule, CPF: coils of the polar filaments, Ref: Reference. ^a^ Mean ± SD; ^b^ Minimum–maximum; ^c^ Mean; ? Data not available.

## Data Availability

All the datasets generated or analyzed during this study are included in this published article.
